# Fine-scale variation in microclimate across an urban landscape shapes variation in mosquito population dynamics and the potential of *Aedes albopictus* to transmit arboviral disease

**DOI:** 10.1371/journal.pntd.0005640

**Published:** 2017-05-30

**Authors:** Courtney C. Murdock, Michelle V. Evans, Taylor D. McClanahan, Kerri L. Miazgowicz, Blanka Tesla

**Affiliations:** 1 Department of Infectious Diseases, College of Veterinary Medicine, University of Georgia, Athens, Georgia, United States of America; 2 Odum School of Ecology, University of Georgia, Athens, Georgia, United States of America; 3 Center for Tropical and Emerging Global Diseases, University of Georgia, Athens, Georgia, United States of America; 4 Center for the Ecology of Infectious Diseases, Odum School of Ecology, University of Georgia, Athens, Georgia, United States of America; 5 Center for Vaccines and Immunology, College of Veterinary Medicine, University of Georgia, Athens, Georgia, United States of America; 6 University of Georgia Riverbasin Center, University of Georgia, Athens, Georgia, United States of America; 7 Mathematics, University of Arkansas Little Rock, Little Rock, Arkansas, United States of America; Santa Fe Institute, UNITED STATES

## Abstract

Most statistical and mechanistic models used to predict mosquito-borne disease transmission incorporate climate drivers of disease transmission by utilizing environmental data collected at geographic scales that are potentially coarser than what mosquito populations may actually experience. Temperature and relative humidity can vary greatly between indoor and outdoor environments, and can be influenced strongly by variation in landscape features. In the *Aedes albopictus* system, we conducted a proof-of-concept study in the vicinity of the University of Georgia to explore the effects of fine-scale microclimate variation on mosquito life history and vectorial capacity (VC). We placed *Ae*. *albopictus* larvae in artificial pots distributed across three replicate sites within three different land uses–urban, suburban, and rural, which were characterized by high, intermediate, and low proportions of impervious surfaces. Data loggers were placed into each larval environment and in nearby vegetation to record daily variation in water and ambient temperature and relative humidity. The number of adults emerging from each pot and their body size and sex were recorded daily. We found mosquito microclimate to significantly vary across the season as well as with land use. Urban sites were in general warmer and less humid than suburban and rural sites, translating into decreased larval survival, smaller body sizes, and lower per capita growth rates of mosquitoes on urban sites. Dengue transmission potential was predicted to be higher in the summer than the fall. Additionally, the effects of land use on dengue transmission potential varied by season. Warm summers resulted in a higher predicted VC on the cooler, rural sites, while warmer, urban sites had a higher predicted VC during the cooler fall season.

## Author summary

Environmental factors influence the dynamics of mosquito-borne disease transmission. Most models used to predict mosquito-borne disease transmission incorporate climate data collected at coarser scales than mosquitoes typically experience. Climate conditions can vary greatly between indoor and outdoor environments, and are influenced by landscape features. We conducted a field experiment with the Asian tiger mosquito to explore how microclimate variation across an urban landscape affects mosquito life history and potential to transmit arboviruses, like dengue. We demonstrate that climate conditions captured by weather stations do not reflect relevant mosquito microclimate, and that subtle variation in mean and diurnal ranges of temperature and relative humidity can lead to appreciable variation in key mosquito / pathogen traits that are important for transmission. Our results have implications for statistical and mechanistic models used to predict variation in transmission across seasons, regions, and land uses, but also for building in realistic environmental variation in laboratory work on mosquito-pathogen interactions. Finally, the variation in microclimate we observed across land use was subtle; likely because our study site is a relatively small city. Nevertheless, these translated into considerable differences in mosquito traits and dengue transmission potential, suggesting these effects could be much larger in more expansive cities.

## Introduction

Epidemics of dengue, chikungunya, and Zika are spreading explosively through the Americas creating a public health crisis that places an estimated 3.9 billion people living within 120 different countries at risk. This pattern began with the growing distribution of dengue virus (DENV) over the past 30 years, infecting an estimated 390 million people per year. More recent invaders, chikungunya (CHIKV) and now Zika virus (ZIKV), are rapidly following suit. CHIKV emerged in the Americas in 2013 and has caused 1.8 million suspected cases from 44 countries and territories (www.paho.org) to date. In 2015, outbreaks of Zika virus (ZIKV) have spread throughout the Americas, resulting in over 360,000 suspected cases, with likely many more going unreported (www.paho.org).

Temperature is one of the key environmental drivers influencing the dynamics and distribution of these diseases [1–10]. Variation in temperature can profoundly impact mosquito population dynamics [11], mosquito life history traits [12–18], mosquito immune responses [19–22]), and measures of parasite / pathogen fitness (prevalence, titers, and the extrinsic incubation period) [1, 10, 23, 24]. In addition to environmental temperature, variation in precipitation [25–27] and relative humidity [28] also drive vector-borne disease transmission. Most statistical and mechanistic models used to predict mosquito borne disease transmission incorporate climate drivers of disease transmission by utilizing environmental data collected from general circulation weather models [1, 29–32], down-scaled weather data [33], outdoor weather stations [34, 35], or remotely sensed land surface temperature data [36–38]. While working with these data is methodologically tractable, mosquitoes do not experience environmental variation at such coarse scales [39, 40]. Temperature and relative humidity can vary greatly between indoor and outdoor environments [41, 42], and can be influenced strongly by variation in landscape features such as density of housing, housing material, vegetation cover, impervious surface cover, waste heat generation, and distance to water [18, 28, 43–48]. Thus, the microclimate a mosquito vector experiences will be dependent upon its dispersal ability (can be < 100 m for some species [49]) and the habitats it visits throughout its life. In addition, many modeling efforts characterize environmental conditions through measures of mean monthly temperature, relative humidity, and precipitation. Yet, there is a growing body of theoretical and empirical work demonstrating that daily fluctuations in temperature, and likely relative humidity, are important for both mosquito and parasite / pathogen traits that mediate transmission [1, 2, 5, 43].

We conducted a semi-field study examining differences in microclimate and mosquito life history traits across a heterogeneous urban landscape to address the above concerns. Specifically, 1) how does mosquito relevant microclimate vary across an urban landscape, 2) how does this variation affect mosquito life history traits, and 3) what are the implications of microclimate variation for vectorial capacity? We investigated these questions in Athens-Clarke Country, GA, focusing on the invasive *Aedes albopictus* (Asian tiger mosquito) system due to its widespread distribution throughout the state [50], as well as its role as an important vector for dengue, chikungunya, and Zika viruses in many parts of the world [51–54].

## Methods

### Site selection

**Fig 1:**
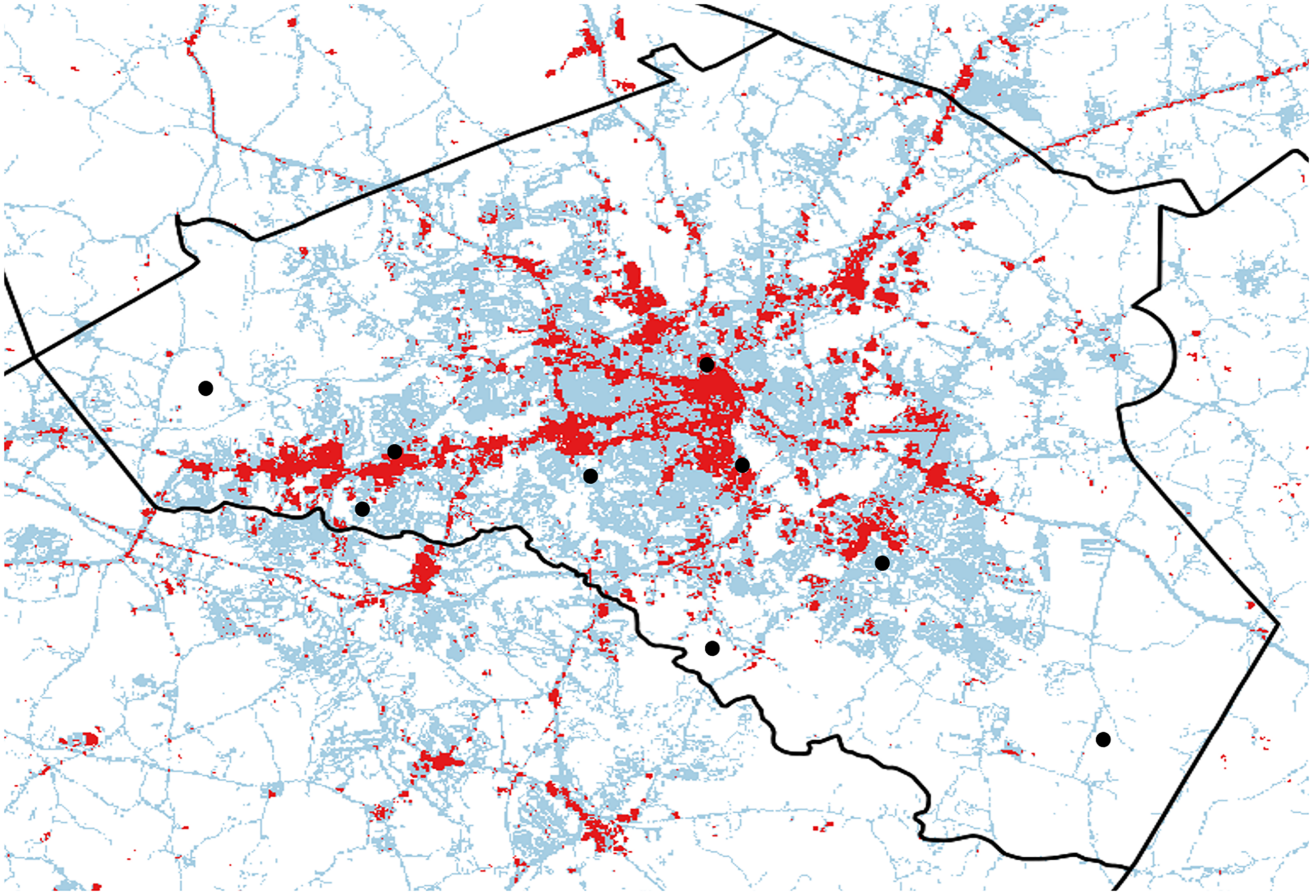
An impervious surface map of Athens-Clarke County, Georgia, U.S. Spatial pixels (30 m^2^) were binned according to proportion of impervious surface, with high, intermediate, and low proportion of impervious surface corresponding to urban (red), suburban (blue), and rural (white) sites, respectively. From this map, we selected three sites (black dots, 30 m^2^) from each land use class for the artificial pot experiments.

We explored microclimate variation across three levels of land use categories characteristic of an urban landscape: urban, suburban and rural. We used an impervious surface map of Georgia generated by the Natural Resources Spatial Analysis Lab at the University of Georgia (http://narsal.uga.edu/glut/data-stats/georgia-impervious-surface-trends) for Athens-Clarke County, Georgia, U.S.A. to distinguish sites into these three land use categories. We defined urban, suburban, and rural sites as those with impervious surface scores within the following binned ranges: 55–100%, 5–50%, and 0%, respectively. We then created a new impervious surface map for Athens-Clarke County and selected three replicate sites within each land use category (Fig 1). Final site selection across Athens-Clarke County was ultimately constrained to sites that we could get permission to access. We did ensure that there was greater than 5 miles between sites, sites were interspersed across the county, and they were of the same area (30 m^2^, Fig 1).

### Larval development experiment

Within each site, we evenly distributed (10 m apart) and staked six black flower pots (Home Depot 480064–1001) in the ground at the base of vegetation (e.g. grass stands, brush, trees) in full shade. Within each pot, we placed a wide-mouth glass bell jar (~1 L, Walmart, 550797441), and added 300 mL of leaf infusion and 30 first instar *Aedes albopictus* larvae. Leaf infusion was made a week prior to the start of the experiment. Live oak (*Quercus virginiana)* leaves were collected from the field and dried in an oven (50°C) for 72 hrs to ensure all water had evaporated from the leaf tissue. We then infused 80 grams of dried leaf material and 3 grams of a 1:1 yeast-albumin mixture in 20 L of deionized water for 3 days prior to use. To monitor variation in larval and adult mosquito microclimate across each site, we added a data logger (Monarch Instruments: RFID Temperature Track-It logger) to each jar and hung a logger (Monarch Instruments: RFID Temperature and Relative Humidity Track-It logger) in vegetation near each jar (~ 3 feet above the ground). Loggers recorded instantaneous measurements of temperature and relative humidity every 10 min throughout the course of the study. Jars were then screened to prevent escape of emerging adults and a wire cage (8 in x 8 in) with plastic vinyl lining the roof was placed over top and staked into the ground to exclude animals and excess rainfall.

Pots were visited daily and emerged adults were removed. We quantified the total number of adults emerging per day, and recorded the sex and wing length of each emerged adult. Wing length was used as a proxy of mosquito body condition due to its associations with female mosquito fecundity, survival, and vector competence for arboviruses [55–57]. One wing was taken from each individual upon emergence, and measurements were taken from the tip of the wing (excluding fringe) to the distal end of the alula using a dissecting scope and micrometer eye piece. This experiment was conducted twice, once in early summer (June 15-July 14, 2015) and once in the fall (September 7-October 10, 2015) to estimate any effects of season on our response variables.

### Calculating per capita mosquito population growth rates (*r*)

We used the following Eq (1) to calculate per capita intrinsic population growth rates (*r*) for each experimental pot across all sites [58],

$$r=\frac{ln\left(\frac{1}{N_{o}}\sum A_{x}f\left(w_{x}\right)\right)}{D+\left(\frac{\sum xA_{x}f\left(w_{x}\right)}{\sum A_{x}f\left(w_{x}\right)}\right)},$$ (1)

in which *N*_*o*_ represents the initial number of females, *A*_*x*_ the number of adult females emerging per day *x*, *w*_*x*_ the mean wing length of females emerging on day *x*, *D* the delay between female emergence and first oviposition, and *f(w*_*x*_*)* predicts the numbers of female offspring produced by females of a given wing size. Because 1^st^ instar mosquito larvae cannot be reliably sexed, and 30 1^st^ instar larvae were deposited in each experimental pot, we assumed *N*_*o*_ to be 15 females. We also assumed *D* = 14.2 days for *Ae*. *albopictus* [58]. We used the following linear function, *f*(*w*_*x*_) = -121.240 + 78.02*w*_*x*_, to describe the relationship between mean wing size and fecundity [59]. While other relationships between wing length and egg production exist [60–62], we chose the relationship characterized in [59] for two reasons. First, this study used variation in mean temperatures to generate *Ae*. *albopictus* adults of different sizes, which ensured we were predicting egg production from variation in wing lengths generated from an environmental variable we allowed to vary in our study. Second, other environmental manipulations (e.g. density, food availability) can alter mosquito body condition or teneral reserves relative to variation in environmental temperature [56], potentially resulting in different relationships between wing length and egg production.

### Statistical analysis

To estimate the effects of microclimate and land use on the larval development and mosquito emergence rates, we used Cox proportional hazard models (R version 3.3.0, package ‘survival’) to assess how these predictors influenced probability of mosquito emergence across pots in each season (*summer* and *fall*). Each model included land use (*rural*, *suburban*, and *urban*) and the following microclimate covariates (*daily temperature mean*, *minimum*, and *maximum* in each experimental pot and average *daily relative humidity mean*, *minimum*, and *maximum*) as predictor variables. Additionally, to control for correlated observations, pot was included as a cluster variable in the analysis. We achieved our final models by using a multidirectional stepwise selection method designed to minimize Akaike Information Criterion (AIC) [63]. All predictors included in final models were checked by using chi-squared tests to verify the assumption that the hazard functions are proportional over time for each strata was upheld. Influential observations and nonlinearity were investigated by removing one observation for each covariate and observing how much the regression coefficients changed and plotting the Martingale residuals (the difference between the observed and expected number of events at each time interval) against each covariate, respectively.

We used general linear mixed effects models (JMP Pro 12.1.0) to investigate the effects of season (*summer* and *fall*), land use class (*rural*, *suburban*, and *urban*), and the interaction on metrics of larval microclimate (*average daily mean*, *minimum*, *and maximum temperature* in each experimental pot and *average daily mean*, *minimum*, *and maximum relative humidity*), mosquito body size upon emergence (*wing size*), the per capita mosquito population growth rate, *r*, and overall transmission potential. Experimental pot was nested within site as a random factor within each model. Sex, and the interactions with season (*sex x season*) and land use (*sex x land use*), were also included as predictors in the model with wing size as the response variable. Model fit and assumptions of normality were assessed by plotting model residuals and quantile plots, and Tukey HSD adjusted pairwise comparisons were run to compare differences across land use groups and for any significant interactions. Boxplots of raw data for each of our response variables are included in additional supplementary information files (S1, S2 and S3 Figs).

### Estimating the effects of season and land use on transmission potential

To estimate how variation in relevant microclimate across different land uses and season might influence the ability of *Ae*. *albopictus* to transmit arboviruses, we used a dengue-specific vectorial capacity framework. Vectorial capacity (*VC*) is a mathematical Expression (2) that describes the daily rate at which future infections arise from one infectious mosquito [10, 64–66]:

$$VC=\frac{ma^{2}bce^{-\mu /EIR}}{\mu },$$ (2)

where *m* represents vector density, *a* is the daily probability of a female mosquito taking a blood meal, *μ* is the daily probability of adult mosquito mortality, *b* is the probability of transmission from an infectious human to a susceptible mosquito, *c* is the probability of transmission from an infectious mosquito to susceptible human, and *EIR* is the extrinsic incubation rate of a pathogen. The density of mosquitoes (*m*) was estimated with the following Eq (3):

$$m=\frac{EFDp_{EA}MDR}{\mu^{2}},$$ (3)

with *m* being comprised of the number of eggs laid per female per day (*EFD*), the egg to adult survival probability (*p*_*EA*_), the development rate of larvae (*MDR*), and adult daily probability of mortality (*μ*).

We incorporated both parameter estimates derived from observations in our semi-field experiment (*p*_*EA*_, *MDR*, and *EFD*) with estimates of parameters from the literature (*a*, *b*, *c*, *EIR*, *μ*) to calculate the effects of season and land use on vectorial capacity. From our survival analyses in our semi-field experiment, we estimated the probability of egg to adult survival (*p*_*EA*_) and the mosquito development rate (*MDR*) as the maximum proportion of adult females emerging across each site and the slope of the inflection point of the cumulative emergence curves, respectively. We also estimated the number of eggs laid per female per day (*EFD*) by taking the number of expected eggs laid per gonotrophic cycle based on body size, using the linear relationship between eggs laid and wing length (y = 78.02x-121.24) [59]. Because there is uncertainty in our estimates of *EFD* that is introduced from the allometric relationship of wing size and egg production [59], we used Monte Carlo simulations to incorporate this uncertainty into our estimates of vectorial capacity. We first generated a variance-covariance matrix from the linear regression of wing size and egg production to generate a contour using the *mvtnorm* package in R. This generated a distribution of wing sizes that we can sample to estimate fecundity. For each pot, we used a random sample of 999 wing lengths to calculate associated egg production values. These were then divided by the expected lifespan (1/*μ*) for each pot to generate an *EFD* estimate for each pot. These *EFD* values were then used to estimate a pot-level vectorial capacity, and the average vectorial capacity for each season and land use. Because the number of samples will be artificially inflated by the Monte Carlo permutations, we used the number of sites per season and land use as the true sample size *n* in our standard error calculations.

To estimate the effects of daily mean temperature (*T*) variation across our sites and with season on parameters in vectorial capacity that we did not measure (*a*, *b*, *c*, *EIR*, and *μ*), we used two non-linear functions described in Mordecai et al. [35]. The Briere thermal Eq (4) is used to explain asymmetric, non-linear relationships of traits with mean temperature,

$$x\left(t\right)=cT\left(T-T_{o}\right)\sqrt{(T_{m}-T},$$ (4)

while the quadratic Eq (5) is used to explain symmetric relationships,

$$x\left(t\right)=c\left(T-T_{o}\right)\left(T-T_{m}\right).$$ (5)

In both functions, *T*_*o*_ is the daily minimum temperature, *T*_*m*_ as the daily maximum temperature, and *c* is a fit parameter with values for these parameters taken from Mordecai et al. [35]. In order to estimate potential effects of variation in diurnal temperature ranges across our sites with land use and season on these parameters, we used rate summation [43, 67] Eq (6) defined as

x=∫r(T(t))dt, (6)

where a given trait (*x*) is defined as a rate (*r*) that adjusts instantaneously to temperature (*T*), which in turn is a function of time (*t*). Thus, for each hourly change in mean temperature, we used the Briere Eq (4) to estimate the biting rate (*a*), transmission probabilities associated with vector competence (*b*, *c*), and the extrinsic incubation rate (*EIR*). We used the Quadratic Eq (5) for mosquito mortality (*μ*). Selection criteria for using the Briere vs. the Quadratic curves for each parameter are outlined in Mordecai et al. [35]. We then summed the proportional hourly changes in parameter estimates across the course of the experiment to incorporate the effect of diurnal temperature fluctuation on each parameter estimate.

## Results

### The effect of season and land use on mosquito microclimate

**Fig 2:**
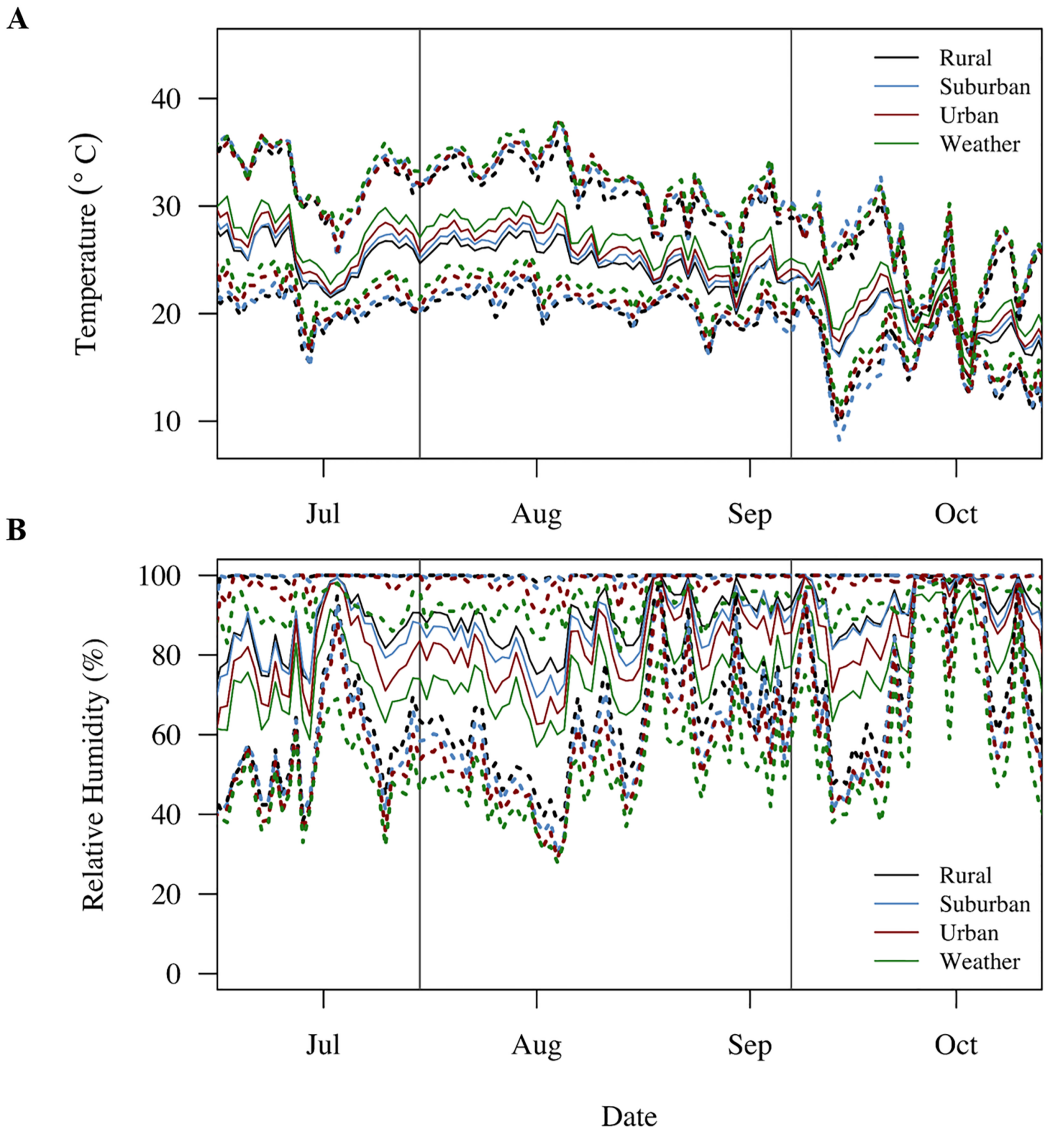
Daily variation in temperature and relative humidity. Ambient mean (solid lines), minimum (lower dotted lines), and maximum (upper dotted lines) daily temperature (**A**) and relative humidity (**B**) were recorded by data loggers across the duration of both experiments on urban (red), suburban (blue), and rural (black) sites and by the local weather station (green) on campus.

We found that the larval microclimate mosquitoes experienced significantly varied with time of season and with land use (Table 1, Fig 2). We did not observe any significant interactions between season and land use, suggesting that the effects of land use on mosquito microclimate were consistent across the summer and fall experiments. Due to larval data logger failure, we were unable to track daily water temperatures across a total of six pots (n = 48 pots) in the summer and one pot (n = 53) in the fall; however, as the failure was equally distributed across treatments, we do not believe this significantly affected our results.

**Table 1:** The effects of season, land use, and potential interactions on daily microclimate.

Factors	*daily mean*			*daily minimum*			*daily maximum*			*diurnal range*		
	d.f.	F	p	d.f.	F	p	d.f.	F	p	d.f.	F	p
*temperature*												
season	1	7329.58	**<0.0001**	1	6851.07	**<0.0001**	1	1215.54	**<0.0001**	1	69.91	**<0.0001**
land use	2	3.74	**0.0307**	2	4.62	**0.0151**	2	78.04	**0.0016**	2	5.40	**0.0076**
season x land use	2	1.05	0.3592	2	3.04	0.0585	2	13.20	0.1955	2	0.09	0.9174
*humidity*												
season	1	774.17	**<0.0001**	1	594.33	**<0.0001**	-	-	-	1	564.93	**<0.0001**
land use	2	53.88	**<0.0001**	2	9.49	**0.0003**	-	-	-	2	4.70	**0.0134**
season x land use	2	2.68	0.0793	2	1.36	0.2726	-	-	-	2	2.49	0.0932

Results from mixed effects models investigating the effects of season (summer vs. fall), land use (rural, suburban, urban), and the interaction on different daily measures of microclimate. Experimental pot nested within site was included as a random factor.

As expected, summer temperatures were on average higher than fall temperatures, with significantly higher daily mean (summer: 26.0°C ± 0.08°C; fall: 20.5°C ± 0.08°C), minimum (summer: 22.4°C ± 0.07°C; fall: 15.6°C ± 0.07°C), and maximum water temperatures (summer: 29.6°C ± 0.12°C; fall: 24.5°C ± 0.12°C). Additionally, experimental pots in the summer were subject to lower daily mean (summer: 82.8% ± 0.30%; fall: 92.8% ± 0.29%) and minimum relative humidity (summer: 55.9% ± 0.63%; fall: 74.8% ± 0.60%). We did not include maximum relative humidity in our analyses because the daily maximum relative humidity across all sites and seasons was consistently close to 100% (Fig 2; S2 Fig). These seasonal differences in daily temperature and relative humidity resulted in summer mosquitoes experiencing a lower diurnal temperature range (summer: 7.3°C ± 0.13°C; fall: 8.9°C ± 0.12°C) and higher diurnal relative humidity range (summer: 43.0% ± 0.63%; fall: 25.0% ± 0.61%) across all sites.

Urban sites were on average warmer than rural sites (Fig 2). Urban sites were characterized by higher daily mean temperatures (Tukey HSD: urban vs. rural, p = 0.0234; urban vs. suburban, N.S.; suburban vs. rural, N.S.) and maximum temperatures (Tukey HSD: urban vs. rural, p = 0.0011; suburban vs. urban, N.S.; suburban vs. rural, N.S.). Interestingly, daily minimum temperatures were similar across suburban and urban sites, with larvae on rural sites experiencing significantly lower daily minimum temperatures (Tukey HSD: rural vs. suburban, p = 0.0123; suburban vs. urban, N.S.; urban vs. rural, N.S.). Urban sites were also significantly drier. Urban sites had lower daily mean relative humidity (Tukey HSD: urban vs. suburban, p < 0.0001; urban vs. rural, p < 0.0001, rural vs. suburban, N.S.) and minimum relative humidity (Tukey HSD: urban vs. suburban, p = 0.0023; urban vs. rural, p = 0.0007). Finally, land use significantly affected fluctuations in diurnal temperature (urban: 8.5°C ± 0.40°C; suburban: 7.9°C ± 0.13°C; rural: 8.0°C ± 0.14°C) and relative humidity (urban: 36.1% ± 0.85%; suburban: 33.2% ± 0.85%; rural: 32.7% ± 0.87%). Urban sites on average experienced wider fluctuations in diurnal temperature (Tukey HSD: urban vs. suburban, p = 0.0023; urban vs. rural, p = 0.0007, suburban vs. rural, N.S.) and relative humidity (Tukey HSD: urban vs. suburban, p = 0.0473; urban vs. rural, p = 0.0183; and suburban vs. rural, N.S.) than suburban and rural sites (but note that the comparison in mean diurnal humidity ranges between urban and suburban sites is only marginally significant).

**Fig 3:**
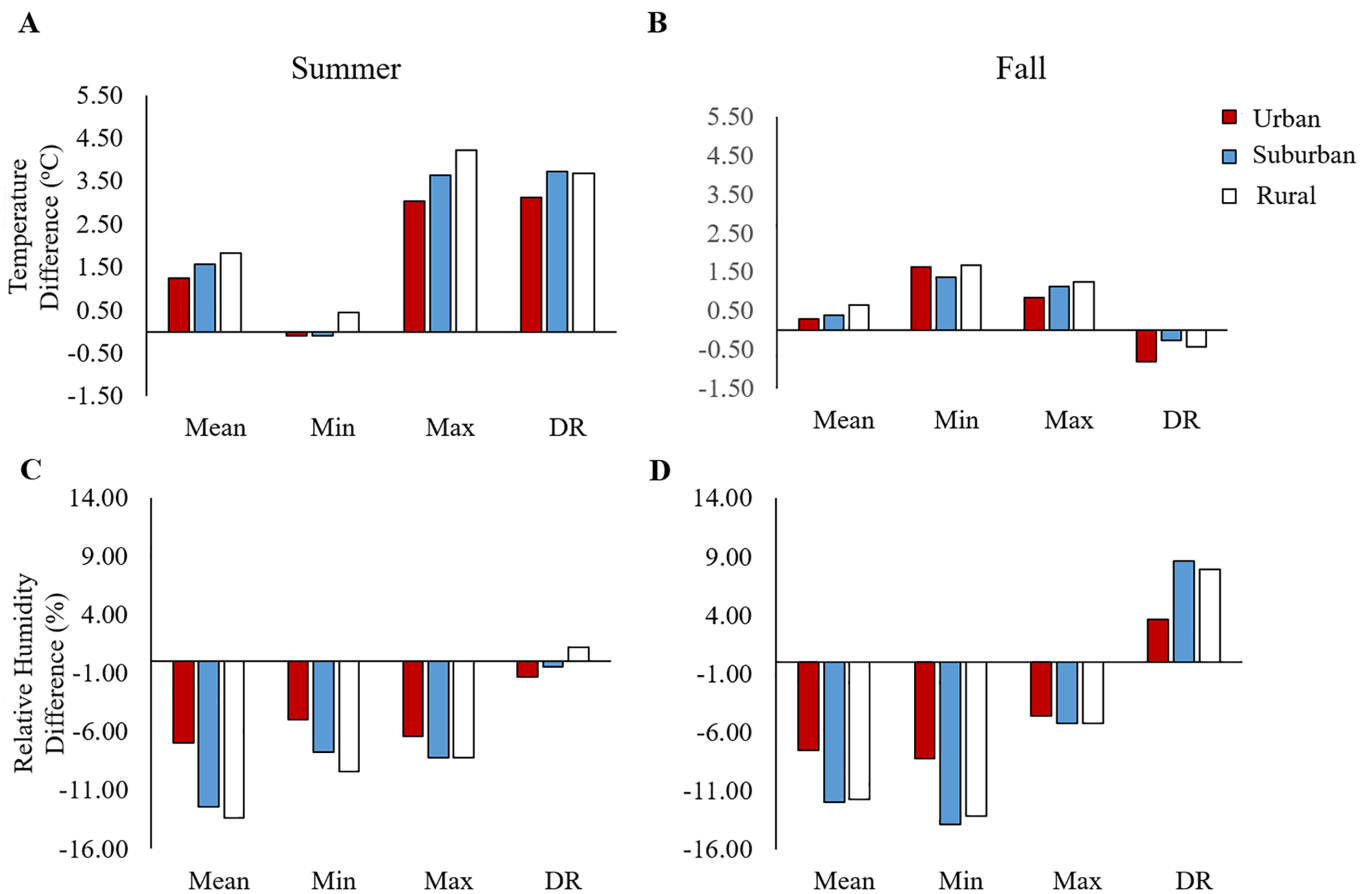
Local weather station data over or under-predict metrics of mosquito relevant microclimate. Differences between daily mean, minimum, and maximum values for temperature and relative humidity recorded by data loggers on urban (red), suburban (blue), and rural (white) sites in the summer (**A**, **C**) and fall (**B**, **D**). Mean and standard errors associated with each land use category reflect estimated marginal means and standard errors from mixed effects models (random factor: pot nested within site) estimating the effects of land use on average daily mean, minimum, and maximum temperatures and relative humidity, while means and standard errors for the weather station data represent data collected from a local weather station at the University of Georgia, Athens GA U.S.A. over the course of each experiment conducted in the summer and fall 2015.

While the daily climate data collected by the local weather station do track the daily variation in temperature and relative humidity recorded by data loggers (Fig 2), the local weather station did not accurately predict daily mean, minimum, maximum, and diurnal ranges of temperature and relative humidity across each land use (Fig 3). Further, the ability of the local weather station to predict urban, suburban, and rural microclimate varied qualitatively across seasons. For example, in the summer, local weather station data over predicted daily mean (by 1.3°C– 1.8°C), maximum, (by 3.0°C– 4.2°C) and diurnal temperature ranges (by 3.1°C– 3.7°C), while under predicting variation in the daily mean (by 6.8% to 13.3%), minimum (5.0%–9.4%), and maximum relative humidity (6.4%–8.2%) across all land uses (Fig 3). In contrast, in the fall, the local weather station was better able to characterize daily mean (a difference of 0.3°C– 0.7°C), maximum (a difference of 0.8°C– 1.2°C), and the diurnal temperature range (-0.8°C to -0.4°C) across these sites. In the fall, like the summer, the local weather station continued to under predict the daily mean, minimum, and maximum relative humidity across urban, suburban, and rural sites. Interestingly, while the difference in relative humidity recorded by the local weather station and our data loggers was minimal in the summer (-1.3%–1.2%), the local weather station in the fall marginally over estimates the relative diurnal humidity range (3.7%–7.8%) in urban, suburban, and rural sites (Fig 3).

### The effect of microclimate, season, and land use on mosquito emergence

**Fig 4:**
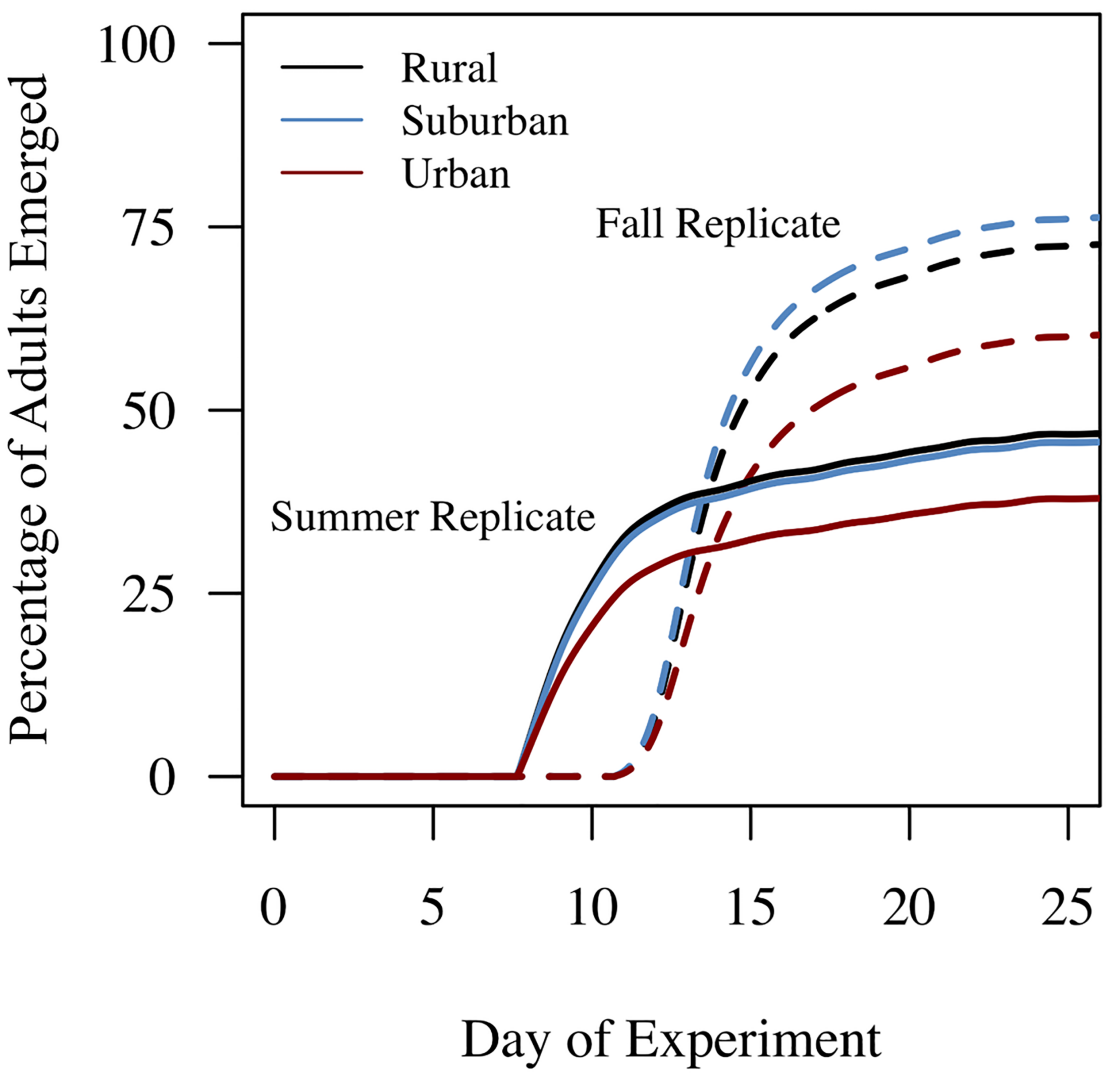
Season and land use both affect the probability of adult emergence. The cumulative percentage of mosquito adults emerging across urban (red), suburban (blue), and rural (black) sites in both the summer (solid lines) and fall (dashed lines).

Overall, larval survival and the number of adult mosquitoes emerging were much higher in the fall than in the summer (Fig 4). Of approximately 1,620 first instar *Ae*. *albopictus* placed into the field during each experiment, we had a total of 318 females and 387 males successfully emerge during the summer replicate and 569 females and 623 males emerge during the fall replicate. Additionally, adults began to emerge at an earlier date in the summer (day 7) than in the fall (day 11). We found significant effects of land use on the likelihood of mosquito emergence in both the summer and fall, with a 44% and 47% decrease in the likelihood of mosquito emergence on urban sites relative to suburban and rural sites (which had similar likelihoods of mosquito emergence), respectively (Table 2). There also was an effect of temperature and relative humidity on mosquito emergence in the summer and fall experiments, but interestingly these effects differed. Mosquitoes developing in the summer experienced an 18% *decrease* in the likelihood of emergence with each 1°C increase in the daily *minimum* temperature and a 7% decrease with each 1% increase in daily *mean* relative humidity (Table 2). In contrast, mosquitoes developing in the fall experienced a 28% *increase* in the likelihood of emergence with each 1°C increase in daily *maximum* temperature and a 19% decrease with every 1% increase in daily *maximum* humidity (Table 2). Together, these results suggest that higher temperatures on urban sites may decrease the likelihood of mosquito emergence through increased larval mortality, and that temperature variation throughout the day has qualitatively different effects on mosquito development and emergence in the summer than the fall.

**Table 2:** The effects of season and land use on mosquito adult emergence.

Factors	*beta*	e^*beta*^	SE	z	p
*summer*					
suburban	0.0818	1.0852	0.1081	0.51	0.6095
urban	-0.4206	0.6567	0.1527	-2.15	0.0313
daily min temperature	-0.1948	0.823	0.0872	-2.13	0.0336
daily mean relative humidity	-0.0693	0.9331	0.0212	-2.64	0.0082
daily min relative humidity	0.0367	1.0374	0.0154	1.6	0.1093
*fall*					
suburban	0.105	1.1107	0.7744	1.08	0.2804
urban	-0.6299	0.5326	0.0972	-2.77	0.0057
daily mean temperature	0.1922	1.2119	0.1007	1.05	0.2932
daily min temperature	-0.236	0.7898	0.0896	-1.57	0.1173
daily max temperature	0.2518	1.2864	0.0769	2.25	0.0243
daily max relative humidity	-0.2066	0.8134	0.0446	-2.93	0.0034

Final model results from a Cox proportional survival analysis estimating the effects of land use (rural, suburban, urban) and microclimate variables (daily mean, minimum, and maximum values of water temperature and ambient relative humidity) on the likelihood of mosquito emergence during summer and fall 2015.

### Effects of microclimate, season, and land use on wing size and *r*

**Fig 5:**
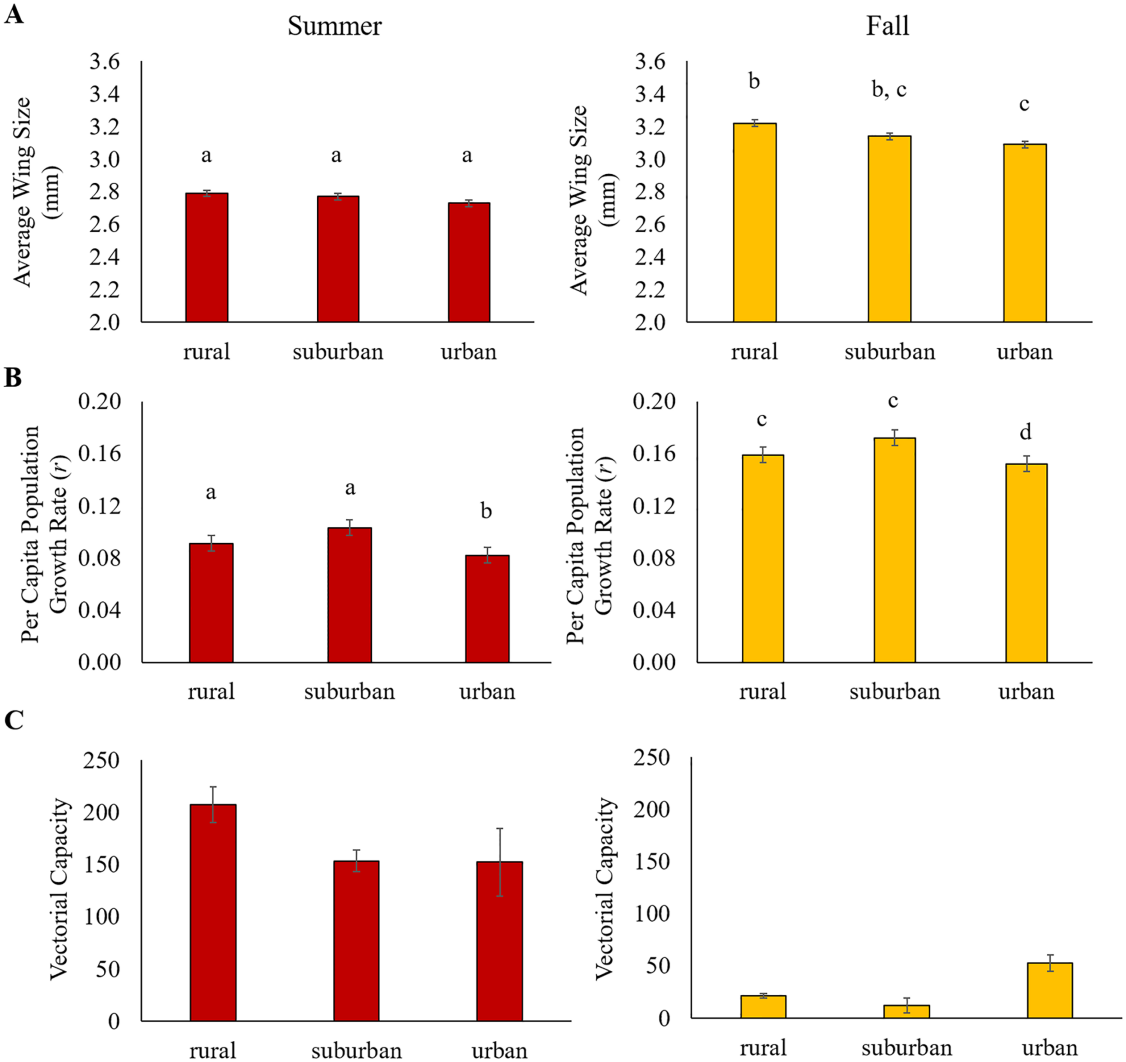
Season and land use have qualitatively different effects on mosquito per capita growth rates and dengue transmission potential. The effects of land use on mosquito body size (**A**), per capita mosquito growth rates (**B**), and relative vectorial capacity, or transmission potential (**C**) in the summer (red bars) and fall (yellow bars). Means and standard errors represent estimated marginal means and standard errors from our mixed model analysis.

We found significant effects of sex, season, and land use on the size of emerging adult mosquitoes (Table 3, Fig 5). Overall, female mosquitoes were larger than male mosquitoes (females: 3.21 mm ± 0.01 mm; males: 2.71 mm ± 0.01 mm). Mosquitoes emerging in the summer were significantly smaller than those emerging in the fall (summer: 2.77 mm ± 0.01 mm; fall: 3.15 mm ± 0.01 mm), and mosquitoes developing on urban sites emerged as smaller adults (urban: 2.91 mm ± 0.02 mm; suburban: 2.96 mm ± 0.02 mm; rural: 3.01 mm ± 0.02 mm) relative to rural sites (Tukey HSD: urban vs. rural, p = 0.0047; urban vs. suburban, N.S.; suburban vs. rural, N.S.). Interestingly, there were significant interactions between season and mosquito sex (*season x sex*) and land use (*season x land use*), suggesting the effects of season on mosquito body size differs for males and females and across land use. For example, female mosquitoes were significantly larger than male mosquitoes (female: 3.21 mm ± 0.01mm; male: 2.71 mm ± 0.01 mm), however this difference in body size was greater in the fall (female: 2.95 mm ± 0.02 mm; male: 2.58 mm ± 0.01 mm; Tukey HSD: female vs. male, p < 0.0001) than the summer (female: 3.46 mm ± 0.01 mm; male: 2.84 mm ± 0.01 mm; Tukey HSD: female vs. male, p < 0.0001). Further, there were no significant effects of land use on mosquito body size in the summer (urban: 2.73 mm ± 0.02 mm; suburban: 2.77 mm ± 0.02 mm; rural: 2.79 mm ± 0.02 mm), however in the fall, mosquitoes emerging on urban sites were significantly smaller (urban: 3.09 mm; suburban: 3.14 mm; rural: 3.22 mm) than those on rural sites (urban vs. rural, p = 0.0003; urban vs. suburban, N.S.; suburban vs. rural, N.S.).

**Table 3:** The effects of sex, season, land use and possible interactions on mosquito wing size, per capita growth rates (*r*), and vectorial capacity.

Factors	*wing size*			*r*			*vectorial capacity*		
	d.f.	F	p	d.f.	F	p	d.f.	F	p
sex	2	2670.26	<0.0001	-	-	-	-	-	-
season	1	1590.73	<0.0001	1	117.14	<0.0001	1	18.76	<0.0001
land use	2	5.48	0.0069	2	3.58	0.0313	2	3.71	0.056
season x land use	1	4.52	0.011	1	0.50	0.6077	1	4.24	0.041
sex x season	1	183.42	<0.0001	-	-	-	-	-	-

Results from a mixed effects model analysis with backward elimination investigating the effects of sex (male, female), season (summer, fall), land use (rural, suburban, urban) and all possible interactions on mosquito wing size, and the effects of season, land use and the interaction on mosquito per capita growth rates (*r*). Experimental pot nested within site was included as a random factor.

Integrating the daily emergence and wing size data into Eq (1), we identified significant effects of season (summer: 0.09 ± 0.004; fall: 0.157 ± 0.004) and land use (urban: 0.115 ± 0.005; suburban: 0.134 ± 0.005; rural: 0.121 ± 0.005) on mosquito per capita population growth rates (*r*, Table 3, Fig 5). Overall, the mosquito per capita growth rate was approximately two times higher in the fall than the summer. Further, the mosquito per capita growth rate was significantly lower on urban sites (Tukey HSD: urban vs. suburban; p = 0.0269; urban vs. rural, N.S.; suburban vs. rural, N.S.).

### The effect of land use and season on arbovirus transmission potential

We found mosquito transmission potential to vector dengue to significantly vary across seasons (Table 3, Fig 5). Transmission potential was higher overall in the summer relative to the fall season. Interestingly, the effects of land use on mosquito transmission potential varied depending on time of season (summer: urban, 152.3 ± 32.2; suburban, 153.6 ± 10.1; rural, 207.6 ± 16.9 and fall: urban, 52.8 ± 8.0; suburban, 12.0 ± 7.1; rural, 21.4 ± 4). The model predicts that during the hot summer, *Ae*. *albopictus* on rural sites have the highest transmission potential relative to suburban and urban sites. In contrast, in the cooler fall, mosquitoes on urban sites were predicted to have the highest transmission potential (Fig 5). Together these results demonstrate fine-scale variation in transmission potential could potentially occur across an urban landscape, and seasonal shifts in microclimate may result in qualitatively different patterns of arbovirus transmission potential with land use.

## Discussion

To date, the majority of studies investigating the effects of urbanization on mosquito population dynamics and disease transmission have been sampling or modeling studies investigating how the distribution and abundance, feeding preferences, and incidence of diseases vectored by different mosquito species vary across land uses [46, 68–77]. In contrast, there have been a handful of experimental studies in the field that mechanistically link observed variation in mosquito traits and metrics of disease transmission to sources of microclimate variation that exist across human-modified landscapes (*Anopheles spp*. [18, 47, 78], *Culex pipiens* [45], *Aedes albopictus* [79]). Our study, in combination with the previous work, demonstrates that relevant microclimate variation in the field (rather than coarser environmental manipulations in the lab) can translate into significant heterogeneity in mosquito life history traits, and ultimately disease transmission potential.

Across both the summer and fall, we observed urban microclimates to be significantly warmer and less humid than non-urban sites, which is reflective of the urban heat island (UHI) effect [80]. This is consistent with other studies showing that urban centers can have different temperature [81–83] and precipitation regimes [84–86] than surrounding areas due to significant modifications to the land-surface structure [44] and increases in the production of waste heat [44]. In other systems, these changes have led to shifts in organism phenology (plants [87–89]), life history (e.g. insect pests, ants, fruit bats [90–93]), and overwintering behavior (mosquitoes [83]), all of which can have significant implications for vector-borne disease transmission [76, 83]. Further, because our study site (Athens, Georgia) is a relatively small city, the observed effects of land use on fine-scale variation in microclimate could be much larger in more expansive cities with greater temperature differentials between urban cores and surrounding areas (3°C-10°C differential [79, 80, 83]).

Despite the subtle effects of land use on mosquito microclimate, we still observed noticeable effects on larval survival, larval development rates, and adult mosquito body sizes, which translated into estimated differences in intrinsic population growth rates and overall transmission potential. This reinforces findings from a diversity of laboratory studies on *Ae*. *aegypti* and *Ae*. *albopictus* demonstrating the effects of relatively large changes in mean temperature [1, 13, 15, 24, 60, 94–100] and diurnal temperature range [1, 7, 101–103] on a diversity of mosquito life history traits (e.g. survival, biting rate, fecundity, larval development, vector competence, and viral extrinsic incubation period). We found mosquitoes developing on urban sites experienced lower survival in the larval environment, emerged as smaller adults, and experienced lower per capita growth rates than on non-urban sites, which could be due to urban sites being in general warmer than non-urban sites. Other similar studies report increases in mosquito development times [45, 79] on urban sites and an increase in adult mosquito emergence [79], which we did not observe.

Surprisingly, different components dictating the diurnal range of temperature and relative humidity were important for larval survival. Overall, in the hot summer, the probability of adult mosquito emergence decreased with higher daily thermal minimums. In contrast, in the cooler fall, increases in the daily maximum temperatures corresponded to increases in the number of adults emerging. Despite having higher average daily thermal maximum temperatures relative to non-urban sites, mosquitoes developing on urban sites still experienced higher larval mortality in the fall. This suggests other unmeasured sources of variation with land use might also influence and have larger effects on larval survival (micro-biotic activity in larval environments, exposure to insecticides, variation in vegetation cover, etc.) on these sites [104, 105]. Variation in relative humidity was also a predictor for the probability of adult emergence across these sites, and like temperature, different metrics of relative humidity were important across different seasons. Interestingly, in both the summer and fall, increases in either the daily relative humidity mean or maximum resulted in proportional decreases in the probability of adult emergence. While an increase in relative humidity has been shown to improve adult mosquito longevity and activity [106–108], it can result in decreases in surface tension of aquatic environments [109], which in turn can increase pupal mortality and decrease the probability of adult emergence in a diversity of mosquito species [110]. To the best of our knowledge, this is the first report of variation in relative humidity affecting the likelihood of larval survival and adult emergence and demonstrates that microclimate variation can have opposing effects on larval and adult traits that are relevant for fitness and transmission.

Variation in daily temperature and relative humidity, as well as the observed variation in mosquito body size with land use and season, could have significant implications for other, unmeasured mosquito traits that are important for arbovirus transmission. For example, variation in both mean temperature and diurnal temperature range in the lab have been shown to impact the daily probability of adult survival (*μ*), female gonotrophic cycles and biting rates (*a*), the number of eggs females produce per day (*EFD*), vector competence (*bc*) and the extrinsic incubation period (*EIP*) for a diversity of mosquito species and pathogens (e.g. *Anopheles* [10, 43], *Culex* [23, 111, 112], *Aedes* [7, 101]). Modeling studies have linked increased precipitation and relative humidity to increased disease incidence (e.g. dengue and malaria) [113–117], likely through the negative effects of low relative humidity (e.g. < 40% relative humidity) on mosquito longevity [107] and activity [108]. Finally, the observed variation in mosquito body size across land use and seasons were consistent with mosquito body sizes reported in other studies and could further compound the effects of microclimate variation on traits like the daily probability of adult survival (*μ*) [118–120], egg production [60–62], and vector competence [95, 121]. For example, observed variation in average wing sizes of mosquitoes across our sites from the summer to the fall (2.7–3.2 mm) could result in individual females producing between 86 and 132 eggs / gonotrophic cycle [59] and result in a 3 fold increase in the probability of dissemination of chikungunya [121].

We used a temperature dependent vectorial capacity equation parameterized for *Ae*. *albopictus* [35] to predict how dengue transmission potential varies across urban, suburban, and rural sites and with season. While the vectorial capacity formula ignores some potentially important sources of variation (e.g. underlying the mosquito-human interaction), it provides a framework for estimating the relative importance of key mosquito / pathogen parameters and the effects of environmental variation on these parameters [1, 43, 122]. Relative vectorial capacity was predicted to be lower in the fall relative to the summer despite the fact that per capita mosquito population growth rates were predicted to be higher in the fall due to increased mosquito survival and egg production associated with increased body sizes. This is due to the negative effect of cooler temperatures on daily probability of mosquito biting (*a*), the extrinsic incubation rate of dengue (*EIR*), and the probabilities of transmission (*b*, *c*) [35], which ultimately result in a smaller proportion of the mosquito population that is infectious and biting at this time of season. We also found arbovirus transmission potential to vary with land use, and the effects of land use on vectorial capacity depended on time of season. These results suggest that the environmental suitability for arbovirus transmission will be dependent upon the shape of the non-linear relationships mosquito and pathogen traits share with temperature, the daily average habitat temperatures and their proximity to the thermal optimum of this non-linear response, and how the effects of daily temperature fluctuation integrate with daily mean habitat temperatures to impact trait performance, and ultimately transmission potential.

This study captures how mosquito life history, population growth rates, and transmission potential respond to variation in microclimate with land use and season. However, there could be variation in other factors that we did not quantify in this study that could ultimately be more important for transmission. Variation in quantity and quality of larval habitat, adult resting habitat, access to hosts, and insecticide application with land use will also likely influence mosquito population dynamics, densities, and transmission potential [73, 123–126]. Further, while environmental conditions shape the potential distribution and magnitude of disease vectors, socio-economic and demographic factors (e.g. variation in human population density, outdoor recreation, housing quality, etc.), human behavior and cultural variation, as well as mosquito feeding preferences will determine the level of human exposure and the realized transmission risk [127, 128]. Thus, even though transmission potential is predicted to be lower in the fall than the summer, seasonal changes in human behavior may result in higher transmission risk in the fall when cooler temperatures encourage more outdoor activity. Likewise, transmission risk may actually be higher in the summer on urban relative to rural sites due to urban sites having higher human population densities. Finally, the replication associated with this study was relatively low, which could introduce uncertainty in our results inherent with small sample sizes.

Most studies that consider the role of climate in vector-borne disease transmission use climate data reported from local weather stations. Our proof of concept study demonstrates that the climate conditions captured by local weather station data do not reflect the microclimates mosquitoes experience, and that subtle variation in mean and diurnal ranges of temperature and relative humidity can lead to appreciable variation in key mosquito / pathogen life history traits that are important for transmission. Greater effort is needed to quantify the activity space mosquitoes occupy and the conditions of relevant transmission environments. This will not only be important for predicting variation in transmission potential and risk across seasons, geographic regions, and land uses, but also for building realistic environmental variation in future laboratory work on mosquito-pathogen interactions.

## Supporting information

S1 Fig Boxplots of mean daily mean, minimum, and maximum temperature and diurnal temperature range data by land use and by season (block 1 = summer; block 2 = fall). (TIF)

S2 Fig Boxplots of mean daily mean, minimum, range of relative humidity by land use and season (block 1 = summer; block 2 = fall). (TIF)

S3 Fig Boxplots of the total number of adults emerging per pot, the wing size of emerging adults, and the estimated intrinsic population growth rate (*r*) by land use and by season (block 1 = summer; block 2 = fall). (TIF)
