# Supplementary figures and images for: Fine-scale variation in microclimate across an urban landscape shapes variation in mosquito population dynamics and the potential of Aedes albopictus to transmit arboviral disease
Source: PLoS Negl Trop Dis. 2017 May 30;11(5):e0005640. doi: 10.1371/journal.pntd.0005640 (PMC5466343; doi:10.1371/journal.pntd.0005640)

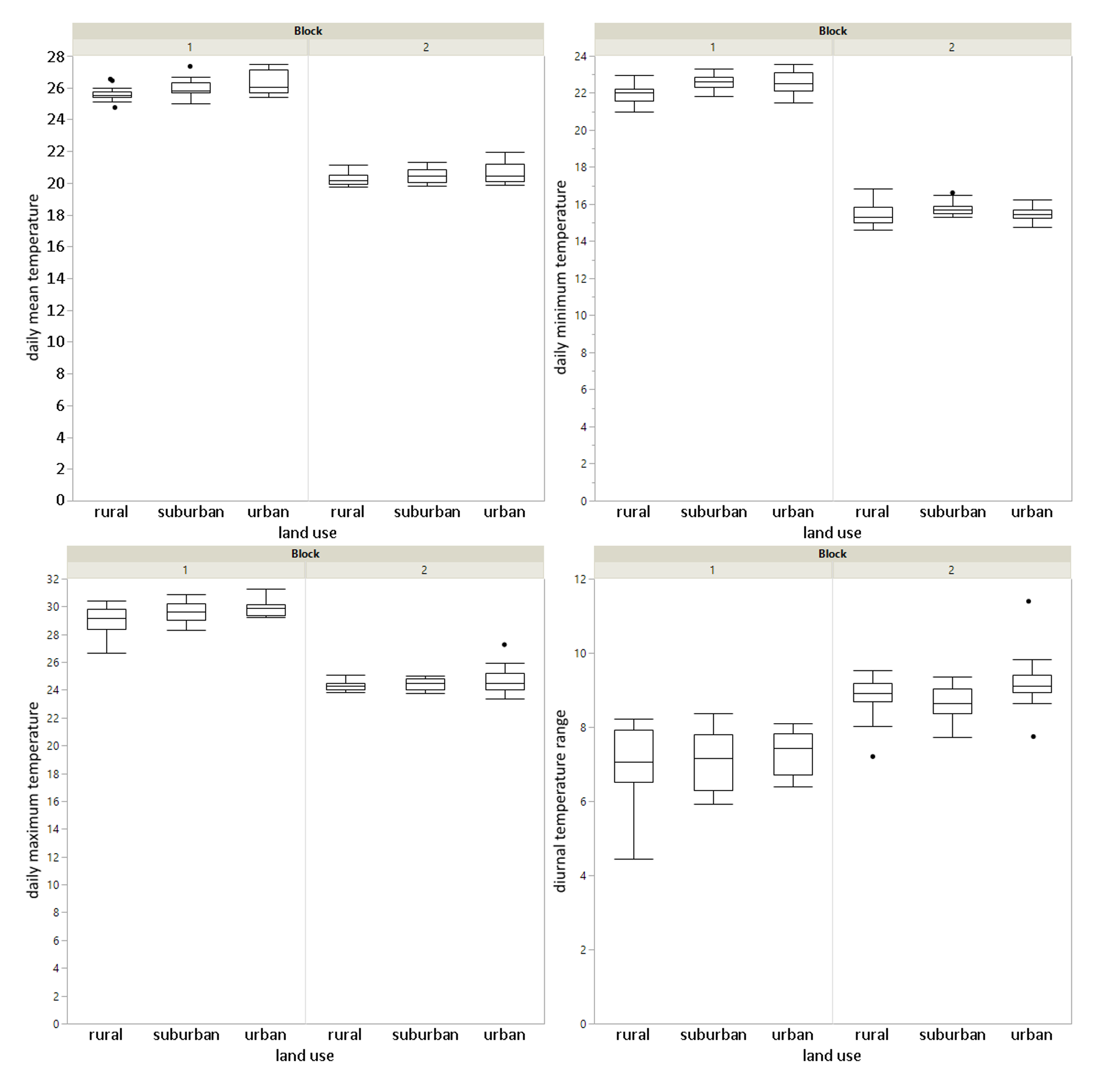

Supplement: S1 Fig — (TIF) [file pntd.0005640.s001.tif]

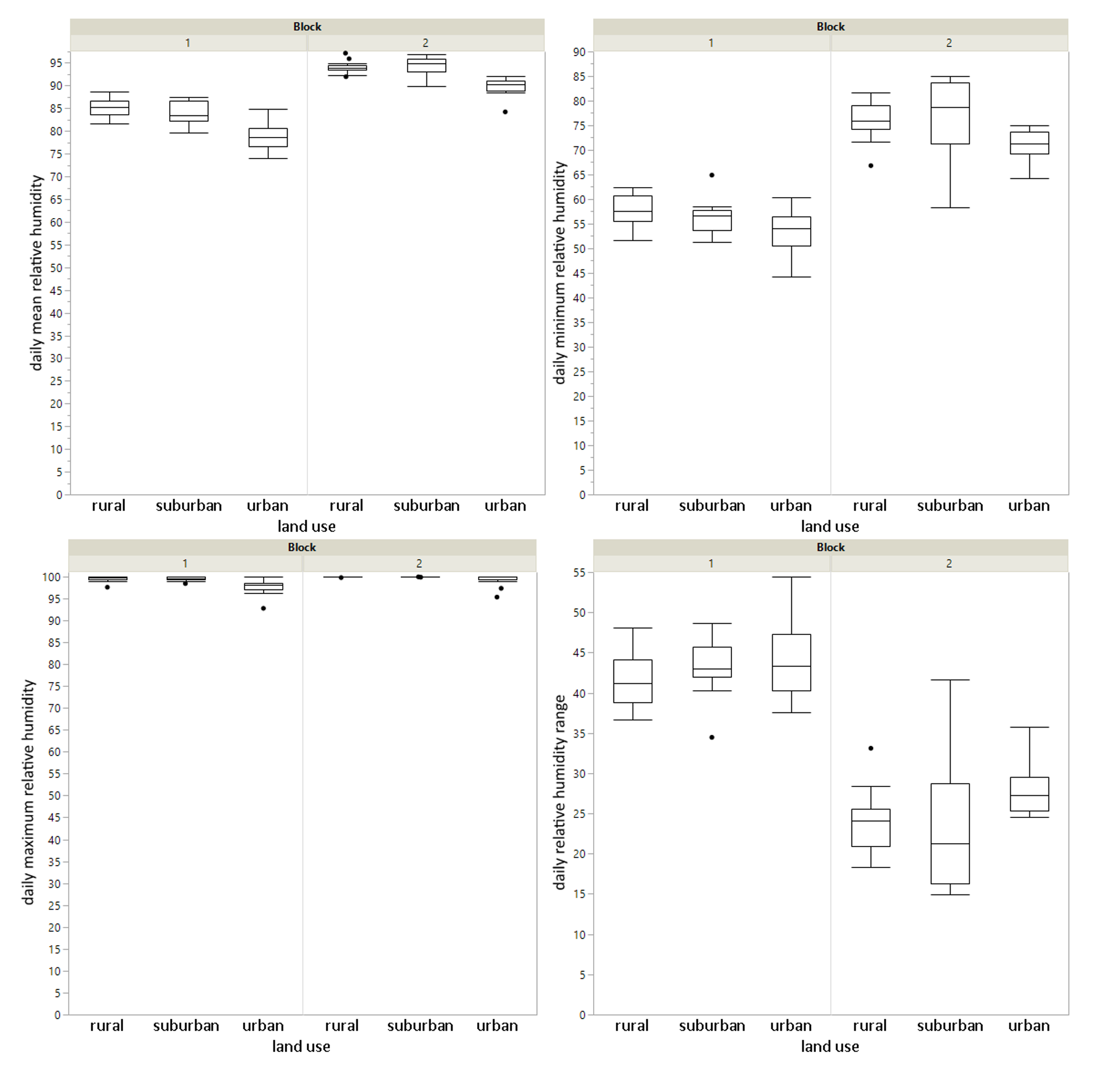

Supplement: S2 Fig — (TIF) [file pntd.0005640.s002.tif]

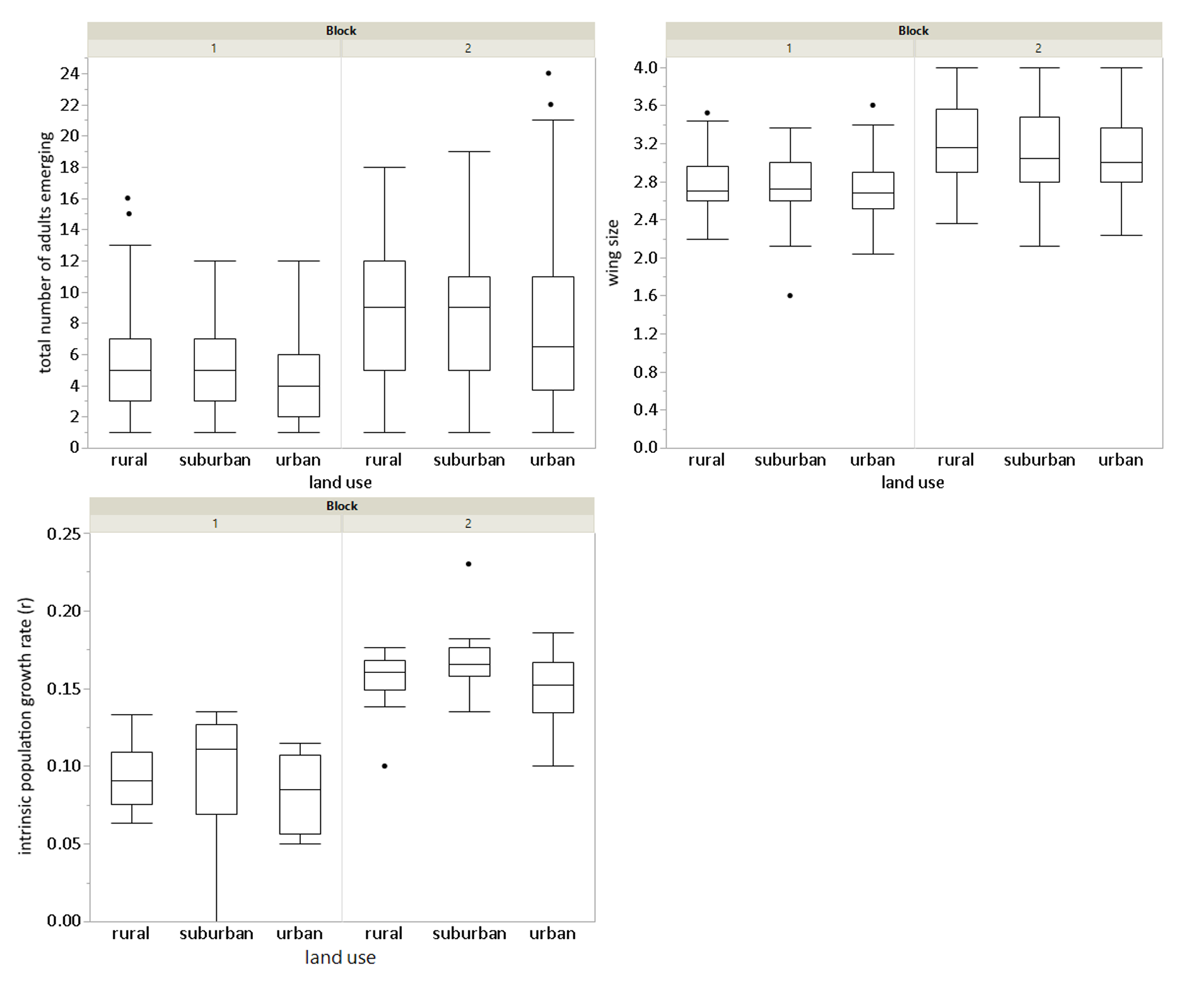

Supplement: S3 Fig — (TIF) [file pntd.0005640.s003.tif]
